# A Postcard From Italy: Challenges and Psychosocial Resources of Partners Living With and Without a Chronic Disease During COVID-19 Epidemic

**DOI:** 10.3389/fpsyg.2020.567522

**Published:** 2020-12-11

**Authors:** Giada Rapelli, Giulia Lopez, Silvia Donato, Ariela Francesca Pagani, Miriam Parise, Anna Bertoni, Raffaella Iafrate

**Affiliations:** Department of Psychology, Family Studies and Research University Centre, Universitá Cattolica del Sacro Cuore, Milan, Italy

**Keywords:** COVID-19, chronic illness, stress, psychological well-being, relational well-being

## Abstract

The new Coronavirus (COVID-19) has been declared a global pandemic by the World Health Organization (WHO). The sudden outbreak of this new virus and the measure of lockdown adopted to contain the epidemic have profoundly changed the lifestyles of the Italian population, with an impact on people’s quality of life and on their social relationships. In particular, due to forced and prolonged cohabitation, couples may be subject to specific stressors during the epidemic. In addition, living with a chronic health condition may add specific challenges to the ones posed by the epidemic itself. The present cross-sectional study aimed to provide a picture of the challenges as well as the resources for both individual and relational well-being of Italian individuals in a couple relationship (*N* = 1921), with a specific attention to the comparison between individuals living with and without a chronic disease. Results showed that people with a chronic disease had lower psychological well-being and more fears and worries about the COVID-19. People with a chronic disease perceived fewer resources than healthy people. Moreover, the challenges are shown to be associated with less psychological well-being and high pessimism about the future. Instead individual, relational, and social resources play a protective role during the pandemic for both healthy and chronically ill people.

## Introduction

**FIGURE 1:**
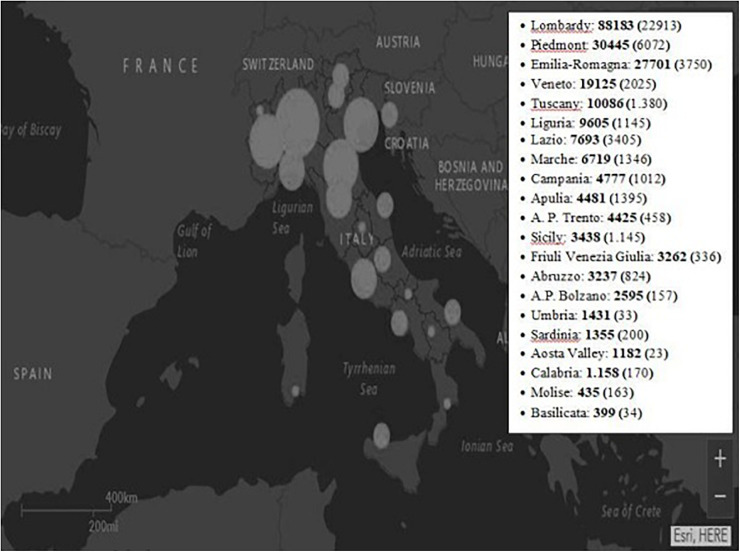
“Map of the situation” by “Sito del Dipartimento della Protezione Civile - Presidenza del Consiglio dei Ministri” is licensed under CC BY-SA 4.0/Modified from original adding a box with number of cases by region. This map shows the total number of cases by region since the outbreak began. In the paranthesis the currently positives as of May 29th, 2020. A.P., autonomous province.

The new Coronavirus (COVID-19) has been declared a global pandemic by the World Health Organization (WHO). In Italy, since the first official case of COVID-19 (February 20th, 2020), a rapid spread of the contagion was reported, making Italy, and especially the North of the country, one of the countries with the highest COVID-19 infection and victim rates (Figure 1). Since March 11th, a strict lockdown was adopted by the Italian government to contain the epidemic: Group activities, social gatherings, outdoor activities were prohibited or strongly limited, businesses that were not regarded as essential were forced to close or -whenever possible- opt for smart-working, etc. Such measures have drastically changed people’s day-to-day life. These changes were essential to contrast the spreading of coronavirus and protect the health system, though they inevitably produced some unintended consequences on people’s lives. Indeed, they profoundly affected people’s quality of life, generating not only changes in lifestyles, social relationships and in the perception of others, but also in the level of stress (Franceschini et al., 2020; Landi et al., 2020; Rossi et al., 2020). In addition to physical and psychological health risks, isolation and loneliness, closure of businesses, organization of home-schooling, economic vulnerability, and job losses were some among the many stressors derived from this emergency (e.g., Crayne, 2020; Di Crosta et al., 2020; Pietrabissa and Simpson, 2020). In fact, pandemic causes psychological consequences on those individuals who are infected by the virus (e.g., Duan and Zhu, 2020), on health professionals (e.g., Barello et al., 2020; Giusti et al., 2020; Vagni et al., 2020a), but also on the non-infected community, because they impact several aspects of social life more generally. In fact, people’s quality of life was profoundly touched by the sudden outbreak of this new virus and by this measure of lockdown (Casagrande et al., 2020; Favieri et al., 2020; Mazza et al., 2020; Zeppegno et al., 2020). Previous studies on COVID-19 reported an influence of both the disease and quarantine measures on psychological well-being (Brooks et al., 2020; Liu S. et al., 2020; Rossi et al., 2020; Xiao et al., 2020), highlighting an increase in anxiety and depressive symptoms and in the perception of lack of control in the general population together with a general decrease in levels of well-being and perception of health in general (Lima et al., 2020).

In this scenario, having a partner, and sharing this emergency with him/her, may be an important protective factor for people’s well-being, as the couple relationship has proved to promote both physical (Koball et al., 2010; Horn et al., 2013) and psychological health (Donato and Parise, 2015; Pagani et al., 2015; Donato et al., 2018a; Pagani et al., 2020). Nonetheless, the couple itself may be subject to specific stressors during the epidemic. Forced and prolonged cohabitation, with no physical space nor time alone to unloading one’s stress and negative emotions, may have put some extra pressure on the couple’s daily life, especially if partners are already engaged in coping with additional stressors (Randall and Bodenmann, 2009).

In particular, living with a chronic health condition may add specific challenges to the ones posed by the epidemic itself (Mazza et al., 2020). Based on currently available information and clinical expertise, people of any age who have serious underlying medical conditions might be at higher risk for severe consequences from COVID-19, thus people living with a chronic disease may have feared to be particularly susceptible to the virus or particularly at risk once infected. Several public and private agencies, in fact, resolved to provide specific guidelines for chronic disease patients in order to respond to their FAQs (e.g., the COVID-19 hotline specific for diabetes patients set up by the Italian Health Ministry). In addition, both the congestion of the health system over several weeks after the virus outbreak and the closing of outpatient services may have made chronic disease patients’ management of the chronic condition as well as their daily life particularly difficult and challenging. The stress pile-up that this segment of the population may have lived with particular intensity might have lasting consequences on their well-being well after the end of the epidemic, which may reverberate on the sanitary system in the long run. On the other hand, people living with a chronic disease may have developed important competences for managing their health as well as stressful health circumstances (Bertoni et al., 2015; Graffigna et al., 2017), which may represent relevant resources to navigate the epidemic period.

The present study aimed to provide a picture of the challenges as well as the resources for both individual and relational well-being of Italian individuals in a couple relationship, with a specific attention to the comparison of individuals living with and without a chronic disease.

Stress is generally recognized as challenging for both individual and relational well-being (Donato and Parise, 2015; Pagani et al., 2015; Donato et al., 2018b; Pagani et al., 2020). In particular, according to Chinese survey data (Liu D. et al., 2020) and the United States Centers for Disease Control and Prevention (Centers for Disease Control and Prevention, 2020), the outbreak of COVID-19 has undoubtedly been a stressful event. The pandemic situation can be highly stressful for individuals at different levels. Changes in the domain of work (job uncertainty, smart-working, work overload), economic worries, and social distancing, on the one hand, and forced cohabitation, on the other hand, may be especially challenging for people’s well-being (Godinic et al., 2020). In addition, fear of contagion is particularly critical for well-being (Mertens et al., 2020; Venkatesh and Edirappuli, 2020), generating anxiety for one’s own personal health and for the health of significant others. Further, the pandemic situation could generate not only concerns for one’s own personal and relational condition, but also concerns for the consequences of COVID-19 at a more global level (i.e., concerns for the world future, concerns for the general community). In light of this, in the present study we focused our attention on three challenges to well-being: global stress, fear of contagion, and worries about the epidemic consequences.

Besides challenges, it is important to identify also those resources that may help individuals to cope with the situation (e.g., Lenzo et al., 2020). In particular, we considered individual, relational, and social resources, which may protect individuals’ well-being. At the individual level, a central concept in understanding how individuals cope with difficulties is their sense of coherence (Antonovsky, 1987). Sense of coherence can be conceptualized as a global orientation that influences the extent to which individuals perceive the world as comprehensible, manageable, and meaningful. Sense of coherence has been found to be linked to well-being and mental health (Anderson, 1998; Eriksson and Lindström, 2007). In a situation in which the foundations of what makes life meaningful and comprehensible have been put at risk, sense of coherence may help maintain well-being. Moreover, individuals in a couple relationship can count not only on their individual coping (Vagni et al., 2020a), but also on dyadic coping strategies. Dyadic coping describes the interpersonal process partners use to jointly cope with stress (Bodenmann, 1997) and is an important factor in maintaining both individual and relational well-being (Donato, 2014; Donato et al., 2015; Pagani et al., 2019).

At the relational level, the capacity to work together as a couple against stress could be a key process in contrasting the negative impact of COVID-19 on partners’ life. Also, at the social level, an important resource could be social support. Social support is an exchange of emotional, informational, or practical assistance with significant others aimed at enhancing the well-being of the recipient (Shumaker and Brownell, 1984). The psychosocial literature, in general, has highlighted the consequences for well-being of being the recipient of supportive acts. In a moment in which social distancing has been imposed as a means of prevention from contagion, perceiving the closeness and the support from one’s family and friends could be protective for well-being. At this regard, we considered both individual (in terms of both psychological well-being and view of the future) and relational well-being (in terms of satisfaction for one’s couple relationship).

In light of these premises, the study had two main goals: (1) To test whether healthy and chronically ill individuals differed in terms of the above challenges and resources. We could expect participants with a chronic disease to be subject to more challenges (i.e., higher levels of stress, fear of contagion, and worries about the epidemic consequences) than participants without a chronic disease. We made no specific predictions with regard to resources (i.e., individual and dyadic coping, family and friend support), as we could expect that, on the one hand, people living with a chronic disease may have developed important competences for managing their health as well as stressful health circumstances, such as a special awareness of their own health, on the other hand, however, their well-being could be more compromised than the one of their healthy counterparts due to their disease. Beyond the higher risk for severe consequences from COVID-19 for people with serious underlying medical conditions, these comparisons might allow us to reveal possible differences between people with and without a chronic health condition also in the psychosocial impact of the COVID-19 epidemic. In addition, we wanted to test whether the impact of a chronic disease on the challenges and resources experienced by individuals in couples depended on whether or not they lived in the North of Italy. The Northern regions of the country, in fact, were those more severely impacted by the epidemic. On the other hand, however, the Northern regions of Italy are also well-known to have the most efficient organization of sanitary services. (2) To analyze the moderating role of health condition (healthy vs. chronically ill) in the associations of the above challenges and resources with participants’ individual and relational well-being (i.e., to test whether healthy and chronically ill individuals differed in these associations as a function of their health condition). This analysis would allow us to identify common and/or specific factors to either contain or promote in order to protect participants’ psychosocial well-being. We might expect a stronger impact of stress, fear of contagion, and worries on the well-being of participants with a chronic disease than in participants without a chronic health condition. As far as resources are concerned, in light of the reasons listed above we made no specific predictions.

## Materials and Methods

### Participants and Procedure

The present study is part of a broader longitudinal research project, titled “The Family at the time of COVID19,” developed by the Family Study and Research University Centre of the Università Cattolica del Sacro Cuore (Milan, Italy) in collaboration with the Human Highway Society. A web-based cross-sectional survey, broadcasted through different platforms and mainstream social-media, was used to collect data. The survey took place from March 30th to April 7th, the period of the national lockdown with constantly growing contagion rates. A brief presentation informed the participants about the aims of the study, and an electronic informed consent was requested from each participant before starting the investigation. The survey took approximately 30 min to be completed. A short questionnaire collected information on some demographic and COVID-19 related information. Standardized questionnaires to evaluate psychological dimensions were administered. To guarantee anonymity, no personal data, which could allow the identification of participants, were collected. Due to the aim of the current research, having at least 18 years was the only inclusion criterion adopted. The study was conducted in accordance with the Ethics Committee of the Department of Psychology of the Università Cattolica del Sacro Cuore. Participants could withdraw from the survey at any moment without providing any justification, and no data were recorded. For the purpose of the present study, we selected people reporting to be in a couple relationship (*N* = 1921). The main demographic characteristics of the sample are shown in Table 1.

**TABLE 1:** Demographic characteristics of the sample.

Variables	Overall sample (*N* = 1921)	Healthy participants (*N* = 1446;76.9%)	Participants with a chronic disease (*N* = 434; 23.1%)
**Gender**			
Female	1281 (66.7%)	952 (65.8%)	300 (69.1%)
Male	640 (33.3%)	494 (34.2%)	134 (30.9%)
**Age**			
18–24 years	12(0.6%)	9 (0.6%)	3 (0.7%)
25–34 years	269 (14%)	224 (15.5%)	40 (9.2%)
35–44 years	656 (34.1%)	527 (36.4%)	114 (26.3%)
45–54 years	617 (32.1%)	458 (31.7%)	144 (33.2%)
55–64 years	272 (14.2%)	176 (12.2%)	92 (21.2%)
Over 65 years	95 (4.9%)	52 (3.6%)	41 (9.4%)
**Italian zone**			
Northern Italy	886 (46.6%)	660 (46.2%)	209 (48.3%)
All other zones	1017 (52.9%)	770 (53.8%)	224 (51.7%)
**Relationship**			
Marriage	1442 (75.1%)	1063 (73.5%)	349 (80.4%)
Cohabiting	479 (24.9%)	383 (26.5%)	85 (19.6%)
**Being a parent**			
Yes	1417 (73.8%)	1058 (73.2%)	327 (75.3%)
No	504 (26.2%)	388 (26.8%)	107 (24.7%)
**Educational qualification**			
Degree or Ph.D.	316 (32.7%)	540 (37.4%)	118 (27.3%)
High school diploma	525 (54.4%)	772 (53.4%)	236 (54.5%)
Secondary school diploma	118 (12.2%)	129 (8.9%)	74 (17.1)
Primary school license	6 (0.6%)	4 (0.3%)	5 (1.2%)

### Measures

The instrument used was a self-report questionnaire composed of the following scales, in addition to socio-demographic data.

#### Fear of Contagion

In order to assess the level of fear of being infected by the coronavirus, participants were asked to express their agreement on a 7-point Likert scale (1 = “not at all” and 7 = “very agree”) to the *ad hoc* item “Are you worried about getting sick of COVID-19 (the disease caused by coronavirus infection)?”.

#### Worries About the Epidemic Consequences

To assess the level of concern about the consequences of the situation connected to the spread of coronavirus, participants were asked to express their agreement on a 7-point Likert scale (1 = “not at all” and 7 = “extremely”) to the *ad hoc* single item “How concerned are you about the current coronavirus situation?”.

#### Stress

To measure their level of stress, participants were presented with a series of statements describing potential sources of stress related to different areas (personal, family or work-related). They were then asked to indicate their degree of stress related to each of these statement on a 5-point Likert scale (1 = “not at all” and 5 = “extremely”). Item examples were “Losing one’s job”; “Managing family life”. The Cronbach’s alpha was 0.87.

#### Individual Coping

Individual coping resources were assessed in terms of participants’ sense of coherence, that is the confidence that one’s environment is predictable and that things will work out as well as it can reasonably be expected (Antonovsky, 1979). Sense of coherence was measured through the Sense of Coherence Scale (SOC; Antonovsky, 1979; Barni and Tagliabue, 2005), which is composed of 13 items rated on a 7-point scale. Items examples are: “Are you surprised by the behavior of people whom you thought you knew well?”; “How often do you have feelings that you’re not sure you can keep under control?”; “How often do you have the feeling that there’s little meaning in the things you do in your daily life?”. We computed a global index of the scale by averaging the 13 items and its Cronbach’s alpha was 0.79. Higher scores represent higher sense of coherence.

#### Dyadic Coping

Dyadic coping is the way partners cope together against stress and was measured by the Dyadic Coping Questionnaire (DCI; Bodenmann, 1997; Donato et al., 2009). We used a selection of 8 items from the original 41-items scale, rated on a 5-point scale ranging from 1 = “never” to 5 = “very often,” that measures positive and negative partner dyadic coping responses (e.g., “My partner proposed practical solutions to the problems that this situation caused”; “My partner accused me of not managing stress well enough”). In the current study, we used the total score that was computed by averaging the 8 items after reversing the negative items: Higher scores represent more supportive dyadic coping responses. The Cronbach’s alpha was 0.71.

#### Family Support

To assess the level of family support, we used the subscale of “The multidimensional scale of perceived social support” (Zimet et al., 1988) focused on the area of family. The four items of this subscale were rated on a 5-point Likert scale (1 = “not at all” and 5 = “very much”). Items examples are: “I can really talk to my family of my problems”; “My family really tries to help me make decision”. The Cronbach’s alpha was 0.93. Higher scores refer to a higher level of support from the family.

#### Friends’ Support

To assess the level of friends’ support, we used the subscale of “The multidimensional scale of perceived social support” (Zimet et al., 1988) focused on the area of friends. The four items of this subscale rated on a 5-point Likert scale (1 = “not at all” and 5 = “very much”). Items examples are: “I can count on my friends when things go wrong”; “I have friends with whom I can share joys and sorrows”. The Cronbach’s alpha was 0.92. Higher scores stand for a higher level of support from friends.

#### Psychological Well-Being

To measure their level of psychological well-being, participants were presented with a series of statements describing possible psychological and physical conditions. They were then asked to indicate their degree of these statement on a 6-point Likert scale (1 = “never” and 6 = “always”) referring to their last week. Item examples were “I felt calm and peaceful”; “I felt discouraged and sad”; “I felt full of energy”. The Cronbach’s alpha was 0.65. Higher scores represent a higher level of psychological well-being.

#### Pessimistic View of the Future

Pessimistic view of the future was measured through the “Dark Future Scale” (Zaleski et al., 2019) which is composed of 5 items rated on a 5-point Likert scale (1 = “absolutely wrong” and 5 = “absolutely true”). Items examples are: “I fear that the problems that worry me now will continue for a long time”; “I am terrified by the thought that I may have to face crises or difficulties in life”. The Cronbach’s alpha was 0.89. Higher scores refer to a more pessimistic view of the future.

#### Relational Well-Being

Relational well-being was measured through one *ad hoc* item. This item (“Overall, how do you rate the relationship with your partner during this period?”), measuring global perception of couple relationship satisfaction, was administered on a 10-point scale (1 = “very negative” and 10 = “very positive”). Higher scores refer to higher relational well-being.

### Data Analyses

Data were analyzed using the software IBM SPSS version 22.0 (SPSS Inc. Chicago, IL, United States). Significance threshold was set at *p* = 0.05. In particular, descriptive statistics were used to summarize the overall and groups’ (i.e., healthy vs. chronically ill) sample characteristics concerning the main variables of the study (Table 2). In order to explore differences among healthy people and people with a chronic disease from different regions of Italy, a series of 2 (Italian zones: Northern Italy vs. Rest of Italy) × 2 (Health status: No chronic disease *vs*. chronic disease) factorial ANOVAs were conducted for the study variables. Finally, a series of hierarchical multiple regression analyses was conducted to test the associations of the challenges (i.e., fear of contagion, worries about the epidemic consequences and stress) and resources (i.e., individual coping, dyadic coping, family support and friends’ support) with the three outcomes of interest (i.e., psychological well-being, pessimistic view of the future, and relational well-being) as well as the moderating role of health condition in the associations between each predictor and each outcome.

**TABLE 2:** Descriptive statistics of all study variables by overall sample and groups.

Areas	Variables	Groups						

		Overall Sample (*N* = 1921)		Healthy people (*N* = 1446)		Chronic disease (*N* = 434)		
		*M*	*SD*	*M*	*SD*	*M*	*SD*	Scale range
Challenges	Fear of contagion	4.67	1.70	4.57	1.69	4.96	1.71	1–7
	Worries about the epidemic consequences	6.08	1.15	6.05	1.16	6.16	1.12	1–7
	Stress	3.47	0.84	3.44	0.85	3.54	0.85	1–5
Resources (individual; dyadic; and social)	Individual coping (SOC)	4.75	1.15	4.79	1.16	4.65	1.12	1–7
	Dyadic coping	3.59	0.71	3.59	0.70	3.59	0.72	1–5
	Family support	3.36	0.97	3.39	0.96	3.23	0.97	1–5
	Friends’ support	3.91	0.88	3.93	0.86	3.82	0.95	1–5
Outcomes	Psychological well-being	3.61	0.88	3.68	0.86	3.43	0.88	1–6
	Pessimistic view of the future	3.49	0.87	3.43	0.87	3.65	0.84	1–5
	Relational well-being	7.86	1.87	7.88	1.85	7.80	1.97	1–10

## Results

### Differences Among Healthy People and People With a Chronic Disease and Among Italian Zones for the Study Variables

A series of two-way factorial analysis of variance (ANOVA) was conducted for each measure. The dependent variable were: Psychological well-being, pessimistic view of the future, and relational well-being, while between-subject factors were: Health status that is the presence or absence of a chronic disease (two levels: 0 = no chronic disease; 1 = presence of a chronic disease), and Italian areas (two levels: 1 = northern Italy; 2 = rest of Italy).

#### Fear of Contagion

The ANOVA showed a significant main effect of Health status [*F*(1,1790) = 16.71, *p* < 0.001, $\eta_{\text{p}}^{2}$ = 0.09]. There was also a statistically significant interaction between the effects of having or not a chronic disease and the Italian areas on fear of contagion [*F*(1,1790) = 3.77, *p* = 0.04, $\eta_{\text{p}}^{2}$ = 0.05]. In particular, only in the central and southern Italy there was a significant effect of health status: People with a chronic disease who lived in central and southern Italy, had significantly more fear of contagion (*M* = 5.15, *SD* = 0.11, *p* < 0.001) than healthy people who lived in the same areas (*M* = 4.57, *SD* = 0.06, *p* < 0.001). In the Northern Italy there was not a significant effect of Health status (Healthy people: *M* = 4.57, *SD* = 0.07, *p* = 0.14; People with a chronic disease: *M* = 4.78, *SD* = 0.12, *p* = 0.14). No significant main effect of Italian areas was found.

#### Worries About the Epidemic Consequences

There was a statistically significant main effect of Italian areas [*F*(1,1859) = 7.69, *p* = 0.01, $\eta_{\text{p}}^{2}$ = 0.06]. In particular, people who lived in northern Italy showed lower levels of worries about the epidemic consequences (*M* = 6.02, *SD* = 0.05) compared to people who lived in the rest of Italy (*M* = 3.19, *SD* = 0.04). The main effect of Health status [*F*(1,1859) = 3.44, *p* = 0.06], and the interaction were not significant [*F*(1,1859) = 1.11, *p* = 0.29].

#### Stress

There was a statistically significant main effect of Italian areas [*F*(1,1859) = 9.14, *p* = 0.01, $\eta_{\text{p}}^{2}$ = 0.06]. In particular, people who lived in northern Italy showed lower levels of stress (*M* = 3.42, *SD* = 0.03) compared to people who lived in the rest of Italy (*M* = 3.56, *SD* = 0.03). There was also a statistically significant main effect of Health status [*F*(1,1859) = 5.06, *p* = 0.03, $\eta_{\text{p}}^{2}$ = 0.06]. In particular, people with a chronic disease showed more stress (*M* = 3.55, *SD* = 0.04) than healthy people (*M* = 3.44, *SD* = 0.02). The interaction was not significant [*F*(1,1859) = 0.10, *p* = 0.75].

#### Individual Coping (SOC)

There was a statistically significant main effect of Health status [*F*(1,1859) = 5.27, *p* = 0.03, $\eta_{\text{p}}^{2}$ = 0.06]. In particular, people with a chronic disease showed lower levels of individual coping, as measured in terms of sense of coherence (*M* = 4.65, *SD* = 0.05), than healthy people (*M* = 4.80, *SD* = 0.03). The main effect of Italian areas [*F*(1,1859) = 1.59, *p* = 0.21], and the interaction were not significant [*F*(1,1859) = 0.22, *p* = 0.64].

#### Dyadic Coping

The main effect of Italian areas [*F*(1,1694) = 0.36, *p* = 0.55], Health status [*F*(1,1694) = 0.03, *p* = 0.87] and the interaction were not significant [*F*(1,1694) = 0.03, *p* = 0.86].

#### Family Support

There was a statistically significant main effect of Italian areas [*F*(1,1859) = 5.08, *p* = 0.02, $\eta_{\text{p}}^{2}$ = 0.06)]. In particular, people who lived in northern Italy showed lower levels of family support (*M* = 3.25, *SD* = 0.04) compared to people who lived in the rest of Italy (*M* = 3.37, *SD* = 0.04). There was also a statistically significant main effect of Health status [*F*(1,1859) = 10.01, *p* = 0.03, $\eta_{\text{p}}^{2}$ = 0.06]. In particular, people with a chronic disease showed lower levels of family support (*M* = 3.23, *SD* = 0.05) than healthy people (*M* = 3.39, *SD* = 0.02). The interaction was not significant [*F*(1,1859) = 2.76, *p* = 0.10].

#### Friends’ Support

There was a statistically significant main effect of Health status [*F*(1,1859) = 2.33, *p* = 0.02, $\eta_{\text{p}}^{2}$ = 0.06]. In particular, people with a chronic disease showed lower levels of friends’ support (*M* = 3.82, *SD* = 0.04) than healthy people (*M* = 3.94, *SD* = 0.02). The main effect of Italian areas [*F*(1,1859) = 2.33, *p* = 0.13] and the interaction were not significant [*F*(1,1859) = 1.91, *p* = 0.17].

#### Psychological Well-Being

There was a statistically significant main effect of Health status [*F*(1,1859) = 28.76, *p* < 0.001, $\eta_{\text{p}}^{2}$ = 0.06]. In particular, people with a chronic disease showed lower levels of psychological well-being (*M* = 3.42, *SD* = 0.04) than healthy people (*M* = 3.69, *SD* = 0.02). The main effect of Italian areas [*F*(1,1859) = 2.60, *p* = 0.11] and the interaction were not significant [*F*(1,1859) = 0.01, *p* = 0.93].

#### Pessimistic View of the Future

There was a statistically significant main effect of Health status (*F*(1,859) = 21.42, *p* < 0.001, $\eta_{\text{p}}^{2}$ = 0.06). In particular, people with a chronic disease showed a more pessimistic perception of the future (*M* = 3.66, *SD* = 0.04) than healthy people (*M* = 3.44, *SD* = 0.02). The main effect of Italian areas [*F*(1,1859) = 0.52, *p* = 0.47] and the interaction were not significant [*F*(1,1859) = 0.06, *p* = 0.80].

#### Relational Well-Being

There was a statistically significant main effect of Italian areas [*F*(1,1859) = 5.86, *p* = 0.02, $\eta_{\text{p}}^{2}$ = 0.06]. In particular, people who lived in northern Italy showed lower levels of relational well-being (*M* = 7.74, *SD* = 1.98) compared to people who lived in the rest of Italy (*M* = 7.96, *SD* = 0.07). The main effect of Health status [*F*(1,1859) = 0.66, *p* = 0.42] and the interaction were not significant [*F*(1,1859) = 0.13, *p* = 0.72].

### Testing the Moderator Effect of Health Status in the Association of Challenges and Resources With Individual and Relational Well-Being

A series of hierarchical multiple regressions were conducted for each outcome (psychological well-being, pessimistic view of the future and relational well-being) to examine (a) the effect of challenges (fear of contagion, worries about the consequences of the epidemic, and stress) and resources (individual: Individual coping; relational: dyadic coping; social: family support and friend support) and (b) the moderating effect of health status in the association between predictors and outcome. To reduce multiple collinearity between variables, the continuous predictors were standardized (Jaccard et al., 1990; Aiken et al., 1991; West et al., 1996; Cohen et al., 2003). Health status was dummy coded (0 = no chronic disease; 1 = presence of a chronic disease) and interaction terms were computed by multiplying the moderator with each of the seven predictors. In the first step, all predictors were included (Challenges: fear of contagion, worries about the epidemic consequences, stress; Resources: individual coping, dyadic coping, family support, friend support). In the second step, interaction terms between each predictor and health status were entered in the analysis. Simple slopes analyses were used to explore significant interactions. In order to control for Type 1 error inflation due to the large number of predictors, a Bonferroni correction for multiple comparisons was employed. Means were considered significantly different when the statistical test’s *p*-value was less than 0.006.

#### Psychological Well-Being

The regression model was significant [*R*^2^ = 0.391, *F*(8,1640) = 131.36, *p* < 0.001]. In particular, as reported in Table 3, among challenges, worries about the epidemic consequences and stress had a negative and significant effect on psychological well-being. On the contrary, the effect of fear of contagion was not statistically significant. Among people’s resources, individual coping, dyadic coping, and family support had a positive and significant effect on psychological well-being. On the contrary, the effect of friends’ support was not statistically significant. The effect of health status was negative and statistically significant. This means that people with a chronic disease reported lower psychological well-being than healthy people. No interaction effects were found.

**TABLE 3:** Testing moderator effects using hierarchical multiple regression on psychological well-being.

Step and variable	*B*	*SE B*	95% CI		β	Partial correlation
**Step 1**						
Fear of contagion	−0.049	0.022	−0.092	−0.007	−0.057	−0.275
Worries about the epidemic consequences	−0.174	0.024	−0.222	−0.127	−0.174*	−0.296
Stress	−0.119	0.022	−0.163	−0.075	−0.137*	−0.362
Individual coping (SOC)	0.372	0.022	0.328	0.415	0.436*	0.518
Dyadic coping	0.064	0.021	0.022	0.106	0.077*	
Family support	0.064	0.021	0.023	0.105	0.075*	0.108
Friends’ support	0.007	0.023	−0.039	0.052	0.008	0.078
Health status	−0.152	0.040	−0.230	−0.074	−0.077*	−0.124
**Step 2**						
Health status*fear of contagion	−0.013	0.045	−0.101	0.074	−0.007	−0.134
Health status* worries about the epidemic consequences	−0.062	0.054	−0.169	0.044	−0.029	−0.170
Health status*stress	0.087	0.046	−0.004	0.178	0.048	−0.141
Health status*individual coping	0.011	0.045	−0.078	0.099	0.006	0.216
Health status*dyadic coping	−0.069	0.044	−0.156	0.018	−0.040	−0.062
Health status*family support	−0.010	0.042	−0.092	0.071	−0.006	0.025
Health status*friends’ support	0.010	0.045	−0.078	0.098	0.006	0.018

CI, confidence interval. *p < 0.006 (p-value after the Bonferroni’s correction).

#### Pessimistic View of the Future

**FIGURE 2:**
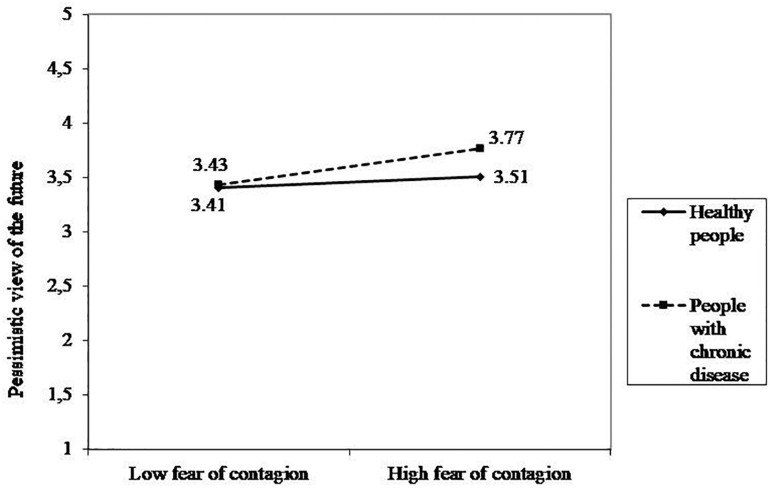
Interaction effect between fear of contagion and health status on pessimistic view of the future.

The regression model was significant [*R*^2^ = 0.332, *F*(8,1640) = 101.989, *p* < 0.001]. In particular, as reported in Table 4, among challenges, worries about the epidemic consequences and stress had a positive and significant effect on participants’ pessimistic view of the future, that is the higher participants’ fear of contagion, worries and stress the more pessimistic their view of the future. On the contrary, the effect of fear of contagion was not statistically significant. Among people’s resources, individual coping had a negative and significant effect on the pessimistic view of the future, which means that the higher participants’ individual coping the more optimistic their view of the future. On the contrary, the effects of dyadic coping, friends’ and family’s support were not statistically significant. The effect of health status was positive and statistically significant. This means that chronically ill people reported a more pessimistic view of the future than their healthy counterparts. The only significant interaction was between Health status and fear of contagion. The simple slope analyses showed a significant positive effect of fear of contagion on the pessimistic view of the future only for people with a chronic disease (healthy people: *b* = 0.05, *p* = 0.09; people with chronic disease: *b* = 0.17, *p* < 0.001; Figure 2). No other significant interaction effects were found.

**TABLE 4:** Testing moderator effects using hierarchical multiple regression on pessimistic view of the future.

Step and variable	*B*	*SE B*	95% CI		*β*	Partial correlation
**Step 1**						
Fear of contagion	0.053	0.023	0.009	0.097	0.061	0.034
Worries about the epidemic consequences	0.152	0.025	0.102	0.201	0.151*	0.088
Stress	0.262	0.023	0.216	0.307	0.301*	−0.017
Individual coping (SOC)	−0.212	0.023	−0.257	−0.167	−0.249*	0.102
Dyadic coping	−0.054	0.022	−0.098	−0.011	−0.065	
Family support	0.006	0.022	−0.036	0.049	0.008	0.076
Friends’ support	0.050	0.024	0.003	0.098	0.059	0.175
Health status	0.130	0.042	0.049	0.212	0.066*	0.018
**Step 2**						
Health status*fear of contagion	0.122	0.047	0.030	0.213	0.068*	−0.017
Health status* worries about the epidemic consequences	−0.036	0.057	−0.148	0.075	−0.017	0.053
Health status*stress	0.004	0.048	−0.091	0.099	0.002	−0.004
Health status* individual coping	0.005	0.047	−0.087	0.098	0.003	0.065
Health status*dyadic coping	0.027	0.046	−0.065	0.118	0.016	−0.007
Health status*family support	0.002	0.044	−0.084	0.087	0.001	0.024
Health status*friends’ support	−0.025	0.047	−0.117	0.067	−0.015	0.083

CI, confidence interval. *p < 0.006 (p-value after the Bonferroni’s correction).

#### Relational Well-Being

**FIGURE 3:**
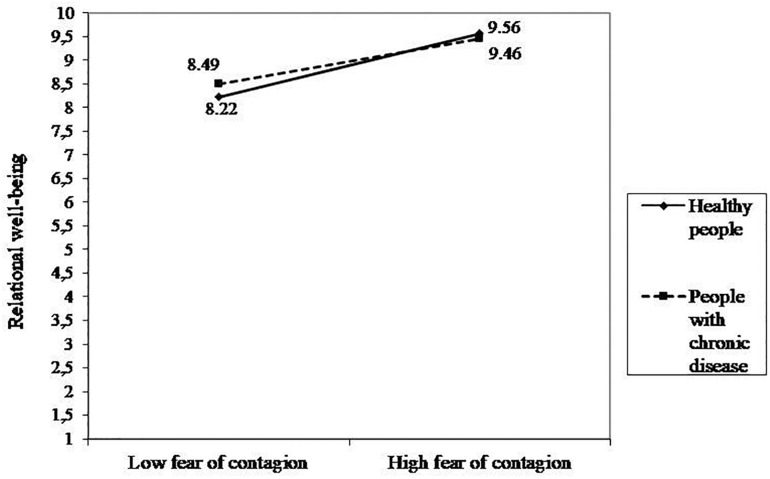
Interaction effect between fear of contagion and health status on relational well-being.

The regression model was significant [*R*^2^ = 0.414, *F*(8,1640) = 144.56, *p* < 0.001]. In particular, as reported in Table 5, the effect of predictors concerning challenges were not statistically significant. Among people’s resources, dyadic coping and friends’ support had a positive and significant effect on relational well-being. The effect of individual coping and family support were not statistically significant. The effect of health status was not statistically significant. The only significant interaction was between Health status and fear of contagion. The simple slope analyses showed a significant positive effect for both healthy people and people with chronic disease, though this association was stronger for healthy individuals than for chronically ill ones (healthy people: *b* = 0.70, *p* < 0.001; people with chronic disease: *b* = 0.50, *p* < 0.001; Figure 3). No other significant interaction effects were found.

**TABLE 5:** Testing moderator effects using hierarchical multiple regression on relational well-being.

Step and variable	*B*	*SE B*	95% CI		β	Partial correlation
**Step 1**						
Fear of contagion	0.070	0.045	−0.019	0.159	0.038	0.309
Worries about the epidemic consequences	0.097	0.051	−0.003	0.197	0.045	0.304
Stress	−0.035	0.047	−0.127	0.056	−0.019	0.469
Individual coping (SOC)	0.110	0.046	0.019	0.201	0.060	−0.383
Dyadic coping	0.963	0.045	0.876	1.051	0.541*	
Family support	0.046	0.044	−0.040	0.131	0.025	0.002
Friends’ support	0.222	0.049	0.126	0.317	0.122*	0.028
Health status	0.093	0.083	−0.070	0.257	0.022	0.117
**Step 2**						
Health status*fear of contagion	−0.197	0.094	−0.381	−0.013	−0.052*	0.196
Health status* worries about the epidemic consequences	0.155	0.114	−0.069	0.379	0.033	0.174
Health status*stress	0.044	0.097	−0.147	0.235	0.011	0.237
Health status* individual coping	0.074	0.095	−0.112	0.261	0.019	−0.174
Health status*dyadic coping	−0.081	0.093	−0.264	0.102	−0.022	0.016
Health status*family support	−0.030	0.087	−0.201	0.142	−0.008	−0.001
Health status*friends’ support	0.050	0.094	−0.134	0.235	0.014	−0.005

CI = confidence interval. *p < 0.006 (p-value after the Bonferroni’s correction).

## Discussion

The present study aimed to provide a picture of the challenges (in terms of stress, fear of contagion, and worries about the epidemic consequences) as well as resources (in terms of individual and dyadic coping as well as social support from family and friends) of Italian individuals involved in a couple relationship. In particular, we analyzed the psychosocial impact of COVID-19 epidemic by comparing healthy people and people with a chronic disease, to underline potential differences between these groups, given the higher risks of contagion and related consequences for unhealthy individuals. In addition, the role of the Italian zones in the above differences was taken into consideration, as the highest number of contagions and deaths due to COVID-19 was registered in Northern Italy. Finally, the study analyzed whether the associations of the above challenges and resources with participants’ individual and relational well-being differed as a function of their health status.

With regard to the first aim of the present study, results have shown that fear of contagion, worries about the pandemic, and the total stress score were higher for people with a chronic disease than for healthy people, although moderate to high levels of these variables were observed for both healthy and chronically ill participants. These results highlight that the COVID-19 epidemic was particularly demanding for people with a chronic disease, in line with recent studies on fear of COVID-19 contagion in people with an oncological disease (Romeo et al., 2020). In fact, for cancer patients, the fear of being infected adds up to the cancer condition, with an explosion of traumatic effects. Focusing on the challenges for healthy and chronically ill people allowed us to detect the different impact of COVID-19 on these populations, showing that people with a chronic disease are more compromised by the situation both physically and psychologically. For these people, in fact, concrete challenges are added at least on two levels. First, the concrete higher risk of being infected by the virus. Secondly, given the impact of the pandemic on ordinary hospital activities, chronically ill people may fear not to be able to manage their own disease and symptoms due to the difficulties in maintaining routine medical treatments or in contacting their own physician. These demands may actually add to the stress and worries of participants living with a chronic disease.

The present findings, moreover, showed an interplay between the health status of participants and their zone of living. More specifically, people with a chronic disease who lived in the central and southern regions of Italy, reported higher levels of fear of contagion than chronically ill people living in the North. Also, the level of stress was higher for people resident in central and southern Italy, despite the study by Casagrande et al. (2020) showed a high level of distress in northern regions. These results, considering the lowest impact of contagion in these zones, were actually unexpected. We could assume that this result may be linked to the fact that southern parts of Italy were impacted by the epidemic at a later time than the North, when the dramatic news about the huge rate of infections and casualties in this region spread out, fomenting serious worries in the rest of Italy, especially in light of the fact that the North was renowned for its higher economic and healthcare system resources as compared to the rest of the country. In fact, the Italian healthcare system has been always decentralized and managed by regional governments and this causes a significant North-South economic divide in favor of the wealthier regions of the North (Cersosimo and Nisticò, 2013) and this situation was further amplified by the outbreak of the COVID-19 (Armocida et al., 2020).

With regard to the area of resources, people with a chronic disease perceived lower individual and social resources than healthy participants, despite having moderate to high levels of these variables. Considering individual resources, people with a chronic disease showed lower levels of sense of coherence compared to the healthy individuals. Seems that the COVID-19 situation, combined with the challenges for the chronically ill population discussed above, decreases in this group the perception of the world and of what is happening around them as understandable, manageable, and meaningful. This is especially critical, considering that individual coping resources were found to be important protective factors in the context of COVID-19 emergency stress (Vagni et al., 2020b). Specific attention should therefore be devoted to sustain chronically ill individual’s coping competences.

With regard to social resources, family and friends’ support levels were high for both groups, but lower for chronically ill participants. It could be that living with a chronic disease, with all the demands that this imposes on individuals’ daily lives, may impair chronically ill people’s social lives. Some evidence exists, for example, that individuals living with a chronic disease experience more loneliness than healthy individuals, even though their social network size and emotional support exchanges does not differ as a function of disease status (Penninx et al., 1999).

Furthermore, it was observed that the level of dyadic coping was moderate to high in both groups and it was independent of the health status. According to the literature on dyadic coping, we could assume that partners cope together in facing a common stressor as shown both on healthy population (Bodenmann, 2005; Donato et al., 2009; Donato and Parise, 2012; Donato et al., 2015; Pagani et al., 2019) and in people with a chronic disease (Bertoni et al., 2015; Rapelli et al., 2020). This specific dyadic skill in both healthy and unhealthy participants emphasizes, firstly, the interdependence of partners’ stress and coping experience and, secondly, the coping process with external stressors as in the case of COVID-19. Given that dyadic coping is a relational competence that partners develop with both minor and major stressors (Bodenmann, 1997; Donato, 2014), both groups may have plenty of occasions to exercise their dyadic coping. Furthermore, we could recognize that Italian couples showed good resources in their couple relationship. The maintaining of a high-quality romantic relationship during times of stress—such as in the case of the COVID-19 pandemic—is very challenging (e.g., Neff and Karney, 2004), as demonstrated also by the divorce rates during COVID-19 lockdown in China and future longitudinal research should examine how partners may adapt to this situation in the long run.

Finally, as far as well-being is concerned, people with a chronic condition showed lower levels of psychological well-being and higher levels of a pessimistic view of the future (Ramírez-Maestre et al., 2019; Rapelli et al., 2020). Besides, relational well-being was similar between the two subgroups and, as previously demonstrated in the literature, a high-quality romantic relationship could be a useful resource to face daily stress (Donato et al., 2009; Donato and Parise, 2012; Donato et al., 2015; Pagani et al., 2019) and also to cope with the pandemic-related stress (Balzarini et al., 2020; Donato et al., in press).

With regard to the second aim of the study, tests of the associations of challenges and resources with individual outcomes showed that worries about the epidemic consequences and stress were negatively associated with psychological well-being, conversely resources area were positively associated with it.

With reference to the resource portfolio of partners, participants’ sense of coherence, that is how meaningful and integrated people view life and the world around them, was positively associated with psychological well-being. This finding confirms what previously found for healthy people (Eriksson and Lindström, 2007) as well as for people afflicted with serious illnesses and disabilities (Anderson, 1998). For example, in chronic patients, sense of coherence was associated with hope and lower symptoms of anxiety and depression (Möllerberg et al., 2019). In a salutogenic perspective, these results suggest that during a pandemic situation it is important to take into consideration also the individual coping strategies and to promote them in order to cope with stress.

Furthermore, dyadic coping and support from family and friends were positively associated with psychological well-being, in line with the literature that explored the supportive role of relationships and their positive effect on well-being among healthy and unhealthy populations (Falconier and Kuhn, 2019).

In particular, dyadic coping was associated with different positive outcomes (Hilpert et al., 2016; Parise et al., 2019), suggesting that having a supportive partner especially during an emergency situation like a global pandemic may alleviate stress, help sharing common difficulties, and improve partners’ psychological well-being. In fact, when an external and shared stressor, like the COVID-19, outbreaks, relying on the partner and on couple skills becomes essential. Moreover, social support could be considered as a crucial protective factor, especially during the COVID-19 epidemic, as previous studies reported that the presence of a social support could help managing a stressful and traumatic event, like for example an illness (Cutrona et al., 2018).

In an opposite direction were the results on participants’ view of their future, in fact worries about the epidemic consequences and stress were positively related to a pessimistic perception of future, the more people reported worries about the COVID-19 situation and the higher their levels of stress, the more pessimistic was their view of the future. Moreover, the significant interaction effect showed that the fear of contagion increased a pessimistic view of the future only for people with a chronic condition. This finding confirms a central role in this emergency situation of fear and uncertainty about the epidemic progression on mental health (Casagrande et al., 2020), as demonstrated also in past virus outbreaks (e.g., Pappas et al., 2009), for people suffering for a chronic disease. In fact, in this population the risk of contagion was higher and the consequences more dangerous. These results indicate that the fear of contagion may crystallize in the present the person with chronic disease, so that the perception of a (good) future is unthinkable. Conversely, higher levels of individual resources protected against a pessimistic perception of the future. In fact, the perception of having a good individual coping was negatively associated with the pessimistic perception of the future, as the person may perceive control over the situation even if it is stressful and a sense of competence in coping with it.

With regard to participants’ relational well-being, interestingly, the significant interaction showed that the association of fear of contagion with relational well-being of participants was positive and significant for both healthy and chronically ill participants, though it was weaker for people with a chronic disease. This result seems to suggest that during COVID-19, when social relationships were reduced due to strict isolation and social distancing, the more people were afraid of an external threat like the virus, the more they perceived their couple relationship as satisfactory. This apparently counter-intuitive finding could have at least two explanations: first, people may need close support and to perceive it positively to cope with the threat of the virus; secondly, the unusual closeness and time spent together as a couple due to the lockdown could allow participants to increase their marital quality. Furthermore, if, on the one hand, fear of contagion precludes the perception of an optimistic future for chronically ill people as previously discussed, on the other hand, it reveals its “generative” effect by activating relational resources and becoming an occasion to test the strength of the couple’s relation, both for the sick and the healthy participants (Donato et al., in press). In fact, the fear of contagion seems to strengthen the couple’s bond, the partners are more united to fight a common enemy (the virus) and therefore more satisfied with their relationship.

With reference to resources, dyadic coping and friends’ support were significantly associated with participants’ relational well-being and in a positive direction. In time of COVID-19 in fact, perceived good support from the partner or friends (but not from family) was associated with a positive perception of participants’ relational well-being. In light of the importance of the quality of the couple relationship for people’s physical and mental health (Donato and Parise, 2015; Pagani et al., 2015; Donato et al., 2018b; Pagani et al., 2020), these findings highlight several key avenues professionals could take in order to sustain and promote both healthy people’s and chronically ill individuals’ couple relationships.

The results of the present study also underline the importance of taking a privileged look in the category of subjects with chronic disease, most affected by the current health emergency. In particular, the present findings have implications for the development of target interventions for the most vulnerables’ needs, which take psychological and social (as well as medical) aspects into consideration. In particular, interventions could pay attention to activities devoted to reduce stress and enhance individual and dyadic coping skills of chronic patients as well as promote social support (e.g., through the activation of online groups).

To the best of our knowledge, our study is the first to explore the psychosocial effects of the COVID-19 emergency in the Italian population focusing on participants in couples, with and without a chronic disease. Furthermore, another strength of the study was the focus on both challenges and resources: in an effort to respond to the pandemic it is essential to know what are the most relevant challenges people live as well as the available resources to activate. Resources, moreover, were analyzed in terms of individual, relational, and social ones: in accordance with Bronfenbrenner’s (1979) ecological systems theory, in fact, individuals are embedded within interconnected systems pertaining to the person, his/her relationships, as well as the social and cultural environment in which they live in and taking into account all this different levels, beyond the individual one, is another strength of the present study. The validity and implications of the present findings should be considered in light of some limitations. First, the present study had a cross-sectional design, which means no conclusions can be drawn with respect to the causality of the observed relationships and directionality of relations between variables. Future research should adopt a longitudinal design in order to help address these issues. Second, this study is based on the comparison between two unequally sized groups (unhealthy *vs*. healthy participants), as it was not primarily designed to make such comparison, and no information about the type of chronic disease of our sample was collected. Future research should use comparable samples and collect more information on participants’ chronic conditions. In addition, pre-COVID measures of variables were not possible in the current study. A final limitation has to do with the exclusive reliance on quantitative approach.

Further research could rely on qualitative measures in order to deeply capture the complexity of the experience of living with a chronic disease during a pandemic.

Again, future longitudinal studies could clarify the changes over time and the direction of the associations. Finally, in future research, the directions identified in this study connected to the importance of resources, at an individual, relational, and social level, to face a critical event might be expanded and better analyzed: In fact, the assumption of a salutogenic perspective could promote a better understanding of the situation considering both risks and protective factors and could be useful also for clinicians who have to sustain people with chronic disease. In fact, not only high levels of stress, but also low levels of individual and relational resources could be harmful. To conclude, the COVID-19 epidemic had an impact on different levels and the present results highlight how focusing on both the challenges to face and the resources to sustain may help highlight important avenues for intervention. From a psychological point of view, although chronically ill individuals are especially challenged during this situation and perceive less resources, their resources may be a relevant leverage to use for sustaining their psychosocial well-being in the aftermath of the pandemic.

## Data Availability Statement

The raw data supporting the conclusions of this article will be made available by the authors, without undue reservation.

## Ethics Statement

The studies involving human participants were reviewed and approved by Ethics Committee of the Department of Psychology of the Catholic University of Sacred heart (protocol number 15–20). The patients/participants provided their written informed consent to participate in this study.

## Author Contributions

GR and GL contributed equally to the research. In particular, they contributed to the development of the theoretical framework, to the performance of the statistical analyses, to the analysis of the results, and to the writing of the manuscript. SD, AP, and MP contributed to the development of the theoretical framework and to the writing of the manuscript. AB and RI supervised the writing of the manuscript. All authors contributed to the article and approved the submitted version.

## Conflict of Interest

The authors declare that the research was conducted in the absence of any commercial or financial relationships that could be construed as a potential conflict of interest.
